# Inebriate Asylum

**Published:** 1877-08-20

**Authors:** 


Inebriate Asylum.—The American Association for the cure of inebriates will hold its eighth annual meeting at Chicago, Ill., September 12th, 1877.

